# Quality of Care Delivered to Tuberculosis Patients among Public Hospitals in Central Northeast Ethiopia

**DOI:** 10.4314/ejhs.v30i5.5

**Published:** 2020-09

**Authors:** Balkew Asegidew Tegegn, Betregiorgis Zegeye Hailu, Birhanu Damtew Tsegaye, Gashaw Garedew Woldeamanuel, Wassie Negash

**Affiliations:** 1 Department of Public Health, Debre Birhan Health Science College, Debre Birhan, Ethiopia; 2 HaSET Maternal and Child Health Research Program, Shewarobit Field Office, Shewarobit, Ethiopia; 3 Department of Biomedical Sciences, School of Medicine, College of Medicine and Health Sciences, Wolkite University, Wolkite, Ethiopia; 4 Department of Public Health, College of Health Science, Debre Birhan University, Debre Birhan, Ethiopia

**Keywords:** Quality, TB Care, Ethiopia

## Abstract

**Background:**

Inappropriate Tuberculosis (TB) diagnosis and treatment contributes to unfavorable health outcome among TB patients. Improving quality of healthcare service helps to avert TB related morbidity. Despite these facts, the level of quality of service is not known in the hospitals. Hence, the present study was conducted to assess the quality of care delivered to TB patients among public hospitals.

**Methods:**

A facility-based cross-sectional study was conducted from March 15 to April 30, 2019 in North Shewa Zone, Amhara region, Ethiopia. All TB patients who had follow-up in the hospitals were included. This resulted in the involvement of 82 TB patients. Data was collected by trained data collectors using facility audit, clinical observation checklists, structured questionnaire and in-depth interview. Data was analyzed using SPSS version 20. Binary logistic regression analysis was done to identify the predictors of patients' satisfaction.

**Results:**

In this study, 82 respondents with a mean age of 36.48 (±13.27) years were participated. The mean quality score for structural dimension was 59.5%, and 53.7% of participants were found to be satisfied in outcome dimension. The mean score for process dimension of quality of service were 67.9%. Having TB symptoms were significantly associated with the level of patient satisfaction towards TB care [AOR = 0.217, p = 0.015].

**Conclusion:**

Quality of TB services from structural and outcome dimension were low and higher in process dimension. Thus, careful attention on the quality of services will help to reduce the burden of TB.

## Introduction

Approximately, 95% of all tuberculosis (TB) cases and 99% of deaths occur in developing countries, with the greatest burden in sub-Saharan Africa and South East Asia (1,2). In the middle-income countries, TB has declined relatively rapidly and has begun to loss its status as major public health problem. However, the decline is slow and tuberculosis remains a major public health problem in other developing countries. In these countries, there is a higher frequency of patients with drug resistance TB than in other countries because of a combination of the poor quality of TB care and inappropriate use of anti TB drugs (3). Ethiopia is one of the 27 countries with high multi-drug resistant tuberculosis (MDR-TB) burden. Even if MDR-TB is highly prevalent in retreated TB cases, the prevalence of MDR-TB in newly diagnosed TB patients has been reported to be 2.8% (4).

Literature suggests that poor quality of TB care may result from discrepancies in documentation such as underreporting and gaps in the continuum of care services that the patients receive. Donabedian developed the structures, process and outcomes model as a framework for assessing the quality of healthcare services. Structure consists of physical health facility, medical equipment and staff characteristics. Processes of care involve interactions between users and the healthcare structures, the actual delivery, and receipt of care. Outcomes are consequences of care. Structures as well as processes may influence outcome directly or indirectly (5).

Even if the coverage of direct observation treatment strategy (DOTS) is high, number of people who becomes infected and died due to TB disease is still high (6). It is necessary to view the quality of TB care being rendered to patients with respect to international standards and delivered in an accessible, timely, safe, effective, efficient, and equitable manner. The quality of health care is a public health concern in many countries, particularly in the developing world including Ethiopia (6, 7). The quality of service for TB care is poor and not as expected as of DOTS coverage (8,9). Thus, similar studies are vital for each specific service delivery sites because poor quality service will have diverse causes and it may be organization specific.

Previous studies have identified several health service delivery factors, which have impacts on adherence such as ineffective communication, poorly supervised health staff, incapability of dealing with minor illness and reduced access to TB care units (10). However, there are no previous studies, which examined the factors contributing to poor quality of TB care in the study area. Hence, the objective of this study was to assess quality of care delivered to TB patients among public hospitals in North Shewa zone, Amhara region, Ethiopia. This study will provide important information about the possible determinants of poor quality of TB care. Thus, the result will be important for public health programmers, social workers, policy makers and relevant stakeholders to design and implement effective prevention and control strategies. In particular, it will be important to improve the quality of care delivered to TB patients in the study area.

## Materials and Methods

**Study design and setting**: A facility-based cross-sectional study was conducted from March 15 to April 30, 2019 in public hospitals of North Shewa Zone, Amhara regional state, Ethiopia. North Shewazone has one referral hospital and eight district hospitals that provide treatment for TB patients. These hospitals provide services for about 2,412,308 individuals.

**Study population and eligibility criteria**: The source population was all TB patients who were getting TB treatment in public hospitals of North Shewa zone and those TB patients who were getting TB treatment in the selected public hospitals of North Shewa zone. All TB patients who were getting TB treatment during the study period were included in the study. However, TB patients who were unable to provide information due to serous medical illness were excluded from the study.

**Sample size determination and sampling procedure**: Sample size was determined using single population proportion formula by taking the proportion of level of patient satisfaction (P) as 0.75 from a study conducted in Addis Ababa (11) and with the assumption of 95% confidence interval and 5% margin of error. The source population was less than 10,000 (110 TB patients were obtaining TB treatment in the nine hospitals of North Shewa zone at a time of our desk review). Thus, we have used a correction formula and the final sample size was estimated to be 80. However, the total number of TB patients receiving treatment in the nine public hospitals during the study period was 82 and all of them were included in the study.

During our desk review with TB focal persons in all the nine hospitals of North Shewazone, we realized that the number of TB patients attending these hospitals were not enough to be sampled using proportional allocation. Therefore, all hospitals with their TB patients were included in the study. Moreover, key informants who were working in the hospitals as a TB focal persons were selected purposively.

Level of patient satisfaction was a dependent variable while socio-demographic factors (sex, age, educational status, marital status, income), health service related variables (treatment phase, type of TB, presence of TB symptom, duration of treatment) and others were independent variables. The following operational deintions are used.

**Quality of TB care** was measured according to Donabedian quality measurement model (the result of the three dimensions i.e structural, process and the outcome dimensions).

**Structural dimension** was measured depending on the level of implementation of the WHO-recommended structural inputs by health facilities and the district TB control program. Fourteen ‘yes/no’ questions were used to assess structural dimension. Then, percentage score was calculated and structural quality was classified as: very good (90–100), good (80–89), marginal (70–79), poor (60–69) and very poor (50–59) (2).

**Process dimension** was measured through performance of the health professionals who worked at TB clinic (2). Using observation clinical checklist, the process dimension of the hospitals was investigated. We have used nine indicators (yes/no question) to assess process dimension.

**Patient satisfaction** was measured by using composite variable and mean score of five Likert scale questions reported the result. Thus, ≥ 75% overall satisfaction was considered as good satisfaction and < 75% was considered as poor satisfaction (2).

**Data collection methods and tools**: Different data collection methods and tools were used. Facility audit about the setting, availability of equipment, drugs, supplies, guidelines and trained personnel at the time of survey was conducted. Trained data collectors (clinical nurses) were deployed to assess the structural dimension and to collect information about process indicators using facility audit and clinical observation checklists, respectively. To assess the process dimension of quality of TB care, one TB focal person from each hospital was interviewed about how the treatment is carried out at the time of the survey. Moreover, interviewer administered structured questionnaire was used to collect data from TB patients attending each hospital. Thus, level of patients' satisfaction and outcome dimensions of quality of TB care were assessed using standardized structured questionnaire. These instruments were developed after reviewing various guidelines, particularly the National TB guideline of Ethiopia, thoroughly.

**Data quality assurance and data analysis**: To ensure the data quality, a three days' training was given for data collectors and the supervisor. The questionnaire was translated into Amharic and back into English for consistency. Moreover, pre-test was conducted on 5% of the total sample. The whole data collection process was supervised to check data completeness and consistency.

Data was entered into Epi-info version 7 and exported to SPSS version 20.0 for further analysis. Descriptive statistics was computed, and the results were reported using frequencies and percentages. Moreover, binary logistic regression analysis was performed to identify the determinant factors of patient satisfaction. A p-value <0.05 was considered as statistically significant.

Ethics approval and consent to participate: The study was conducted after ethical letters were obtained from Ethical Review Committee (ERC) of the College of Health Sciences, DebreBirhan University, Ethiopia. Then, permission letter was taken from the zonal health bureau and each hospital'sdirector office. Data was collected after obtaining verbal informed consent from the study participants. To keep confidentiality, codes were used and unauthorized person did not have access to the data.

## Results

**Socio-demographic characteristics**: In this study, 82 respondents were participated with 100% response rate. The mean (± SD) age of the participants was 36.48 (±13.27) years with an age range of 57 years. About 36(43.9%) of the participants were in the age group of greater than 35 years. Moreover, the majority of the respondents, 49(59.8%), were urban dwellers (Table 1).

**Table 1:** Socio-demographic characteristics of the study participants in public hospitals of North Shewazone, Amhara region, Ethiopia, 2019 (n = 82)

Variables		Frequency	Percentage
Age group (years)	< 25	20	24.4
	25 – 35	26	31.7
	> 35	36	43.9
Sex	Male	50	61.0
	Female	32	39.0
Residence	Urban	49	59.8
	Rural	33	40.2
Religion	Orthodox	72	87.8
	Muslim	4	4.9
	Protestant	6	7.3
Marital status	Single	24	29.3
	Married	55	67.1
	Divorced	2	2.4
	Widowed	1	1.2
Educational status	Illiterate	9	11.0
	Elementary	42	51.2
	Secondary	18	22.0
	Certificate and above	13	15.9
Occupation	Government employee	13	15.9
	Private worker	21	25.6
	House wife	12	14.6
	Merchant	9	11.0
	Student	8	9.8
	Others	19	23.2
Monthly income	< 1500	45	54.9
(ETB)	≥ 1500	37	45.1
Family Size	< 5	57	69.5
	≥ 5	25	30.5

Note: ETB = Ethiopian Birr

**Health service related factors**: This study found that about 56(68.3%) of the participants traveled more than 2.5km to reach to TB clinics. Most of the study participants, 64(78%), responded that they accessed the TB clinics easily. This study also revealed that more than two-thirds (64.6%) of the patients were in the 2^nd^ month of TB treatment (Table 2).

**Table 2:** Health service related characteristics of the study participants in public hospitals of North Shewazone, Amhara region, Ethiopia, 2019 (n = 82)

Variables		Frequency	Percent
Distance traveled to	< 2.5km	26	31.7
TB clinic	≥ 2.5km	56	68.3
Means-	On foot	23	28.0
of transportation	Vehicle	59	72.0
Facility visit history	Yes	11	13.4
for TB treatment	No	71	86.6
Able to get TB clinic	Yes	64	78.0
easily	No	18	22.0
Treatment duration	On 1^st^ month	27	32.9
	On 2^nd^ month	53	64.6
	>2 month	2	2.4
Type of TB	Pulmonary positive TB	40	48.8
	Pulmonary negative TB	12	14.6
	Extra-pulmonary TB	29	35.4
	MDR-TB	1	1.2
Treatment category	New	74	90.2
	Retreatment	8	9.8
Presence	Yes	34	41.5
of symptoms	No	48	58.5

Note: TB = Tuberculosis, MDR = Multidrug resistance

## Dimensions of Quality of TB Care

**Structural dimension indicators**: In this study, structural (input) dimension was assessed using standardized facility audit checklist and semi-structured interview guide. In all hospitals, there were separate rooms for TB treatment, availability of recommended anti TB drugs, treatment guidelines, AFB test and standard TB registration book. However, there was no availability of reminder notices (such as how to cover their mouth while coughing and how to use mask) in all of the health facilities. Moreover, it was found that only one of the study hospitals was being piped with clean water to TB clinic. Apparently, the average quality score was calculated for each health facility at the first step, and then, the overall quality of this component was calculated by taking the average of all health facilities. Therefore, the average quality of structural component was 59.5% (Table 3).

**Table 3:** Structural dimension of quality of TB care in public hospitals of North Shewazone, Amhara region, Ethiopia, 2019

Variables	HF_1_	HF_2_	HF_3_	HF_4_	HF_5_	HF_6_	HF_7_	HF_8_	HF_9_	Total
Separate TB room	Yes	Yes	Yes	Yes	Yes	Yes	Yes	Yes	Yes	9
Trained TB care provider	Yes	Yes	Yes	Yes	Yes	Yes	Yes	Yes	Yes	9
Standard/Rx guidelines	No	Yes	Yes	No	No	Yes	Yes	No	No	4
TB poster at TB room	Yes	No	No	No	No	No	No	No	Yes	2
All anti-TB drugs	Yes	Yes	Yes	Yes	Yes	Yes	Yes	Yes	Yes	9
Reminder notices for specific interventions	No	No	No	No	No	No	No	No	No	0
HF is comfortable and safe for TB treatment	No	Yes	Yes	Yes	Yes	Yes	Yes	Yes	Yes	8
Supervisory support in the last 6 months	No	No	Yes	No	No	Yes	Yes	No	No	3
Room with windows	Yes	Yes	Yes	Yes	Yes	No	Yes	Yes	Yes	8
AFB test	Yes	Yes	Yes	Yes	Yes	Yes	Yes	Yes	Yes	9
Clean water	No	No	No	Yes	No	No	No	No	No	1
Comfortable waiting rooms for HE	No	Yes	Yes	No	No	No	Yes	No	Yes	4
Standard TB register	Yes	Yes	Yes	Yes	Yes	Yes	Yes	Yes	Yes	9
HE program on TB	No	No	No	No	No	No	No	No	No	0
Total (available)	7	9	10	8	7	8	10	7	9	
Percentage	50.0%	64.3%	71.4%	57.2%	50.0%	57.2%	71.4%	50.0%	64.3%	**59.5%**

Note:HF = Health facility, AFB = Acid fast bacilli, HE = Health education

The key informants' (TB focal persons') suggestion also indicated that most infrastructures, necessary materials and all anti-TB drugs were available in the majority of the health facilities. These necessary materials and all anti-TB drugs were also observed in our supervision. Moreover, all health facilities had trained health care providers. On the other hand, there were no comfortable waiting areas for health education, reminder notice for specific interventions and clean water source. One TB focal person said: “*All TB patients bring water from their home to take their medication each morning during DOTs phase of treatment because there is no clean water source in our hospital*”.

**Process dimension indicators**: Using observation clinical checklist, the process dimension of the hospitals was investigated. Accordingly, care providers of all health institutions prescribed appropriate regimens and assessed patients' adherence. Moreover, the majority of the health workers did not demonstrate greeting, respectfulness and encouraging attitude to their patients while the patients received their drugs. In contrast, patients were seen in private TB rooms and were participated in decision-making processes of service delivery (Table 4). TB focal persons' (key informants') suggestions also indicated that helath care providers have prescribed appropriate regimens and counseled the patients. However, all TB focal persons said: “*There is a gap of documenting written record of medications, bacteriologic response and adverse reactions of the medications*”.

**Table 4:** Process dimensions of quality of TB care in Public hospitals of North Shewa zone, Amhara region, Ethiopia, 2019

Process indicators	Performing in each hospitals		
	Yes	No	%
Care provider gave greeting for the patient	2	7	2.8
Care providers prescribed appropriate regimen and assessed adherence	9	0	100
Care provider gave medication counseling to the patient	8	1	88.9
Patient were taking their medications by DOT program	8	1	88.9
Maintaining written record of medications, bacteriologic response and adverse reactions	0	9	0
Patient centered approach to administration of drug treatment	8	1	88.9
Care provider gave the patient about appropriate appointment	9	0	100
examination room comfortable to the patients (during examining the Pt)	9	0	100
Care providers demonstrated to the patient how to take medication	2	7	2.8

## Outcome Dimension

**Level of patient satisfaction**: The overall satisfaction score for outcome dimension was 53.7%. The majority of the respondents, 74(90.2%), were satisfied with the respect offered by healthcare providers, and about 70(85%) respondents were satisfied with the adequacy and appropriateness of working hours. About 55(67%) participants were dissatisfied about completeness of information given to the patients regarding treatment and effectiveness of the services (Table 5).

**Table 5:** Level of patient satisfaction with quality of TB care in Public hospitals of North Shewa zone, Amhara region, Ethiopia, 2019

Variables	Level of satisfaction				
	Very Satisfied n (%)	Satisfied n(%)	Neutral n (%)	Dissatisfied n (%)	Very Dissatisfied n (%)
Distance to access HF	4 (4.9%)	66 (80.5%)	3 (3.7%)	9 (11%)	0
Respect offered by HCP	2 (2.4%)	74 (90.2%)	1 (1.2%)	5 (6.1%)	0
Measures taken to assure privacy	78 (95.1%)	3 (3.7%)	1 1.2%)	0	0
Measures to keep confidentiality	2 (2.4%)	77 (93.9%)	3 (3.7%)	0	0
Completeness of information given	1 (1.2%)	55 (67.1%)	7 (8.5%)	17 (20.7%)	2 (2.4%)
Access of HCP as the patients need	2 (2.4%)	47 (57.3%)	2 (2.4%)	30 (36.6%)	1 (1.2%)
Access of HCP to refill medication	4 (4.9%)	44 (53.7%)	4 (4.9%)	29 (35.4%)	1 (1.2%)
HCP welcoming and respect	3 (3.7%)	63 (76.8%)	6 (7.3%)	10 (12.2%)	0
Waiting time	7 (8.5%)	69 (84.1%)	2 (2.4%)	4 (4.9%)	0
Appropriateness of working hours	5 (6.1%)	70 (85.4%)	2 (2.4%)	5 (6.1%)	0
Time spent with HCP	2 (2.4%)	69 (84.1%)	5 (6.1%)	6 (7.3%)	0
Explanation to patient's question	4 (4.9%)	68 (82.9%)	9 (11%)	1 (1.2%)	0
Ability to Dx, treat and care of TB	2 (2.4%)	78 (95.1%)	2 (2.4%)	0	0
Appointment system of follow up	4 (4.9%)	74 (90.2%)	3 (3.7%)	1 (1.2%)	0
Check clinical condition	1 (1.2%)	65 (79.3%)	7 (8.5%)	9 (11%)	0
Uses medical terms	3 (3.7%)	70 (85.4%)	6 (7.3%)	3 (3.7%)	0

Note: HF = Health facility, HCP = Health care provider, Dx = Diagnosis

**Factors associated with patient satisfaction**: First, binary logistic regression analysis was done to see any crude association between the level of patient satisfaction and the various independent variables such as socio-demographic variables and health service related variables. Second, variables with p < 0.25 in bivariate analysis were selected and enrolled into multivariable binary logistic regression analysis. Age, sex, TB symptoms, TB treatment duration and type of TB were the candidates for multivariable analysis. After doing forward logistic regression analysis, having TB symptoms had significant association with level of patients' satisfaction towards TB care [AOR = 0.217, 95% CI = (0.064, 0.743), p = 0.015]. About 79.3% of the patients with TB symptoms were less satisfied than those patients who had no TB symptoms during TB treatment (Table 6).

**Table 6:** Multivariable binary logistic regression analysis of variables associated with patient satisfaction in public hospitals of North Shewa zone, Amhara region, Ethiopia, 2019

Variable		Satisfied	Dissatisfied	COR(95% CI)	AOR(95% CI)
Age (years)	< 25	13	7	1.327(0.427,4.118)	
	25–35	10	16	0.446(0.159,1.252)*	
	>35	21	15	1.00	
Sex	Male	31	19	1.00	
	Female	13	19	0.419(0.169,1.039)	
Presence of TB symptom	Yes	12	22	0.273(0.108,0.688)**	0.217(0.064,0.743)**
	No	32	16	1.00	
Treatment duration	1^st^ month	9	15	1.00	
	2^nd^ month	16	13	2.051(0.680,6.186)	
	>2 month	19	10	3.167(1.026,9.770)	
Type of TB	Pulmonary smear +ve	18	22	1.00	
	Pulmonary smear -ve	6	6	1.222(0.336,4.448)	
	Extrapulmonary TB	20	10	2.444(0.916,6.526)**	

Note:COR = Crude odds ratio, AOR = Adjusted odds ratio

* p- value < 0.25

** p- value < 0.05

*** p- value < 0.001

## Discussion

Treatment of TB is the cornerstone of any National Tuberculosis Program (NTP). Assessing the quality of TB treatment services is important, because it tells us how the health system is performing and leads to improved care. In this study, effort has been made to identify constraints in all components (structural, process and outcome) of TB care using Donabedian's quality assessment model in healthcare.

In this study, the overall quality score of structural components was 59.5%, which indicates that the majority of the studied facilities were structurally poor. The structural quality score found in our study is better than a study conducted in Jimma zone with overall structural quality score of 56% (8). However, it is lower than a study conducted in Sidamazone, which reported 85% overall structural quality score (2). This might be due to discrepancy in the program managers' attention for the program, resource allocation and infrastructure variations.

The current study found weak supervision patterns. Only three public hospitals were supervised in the last 6 months, and the supervision pattern was also unplanned and lacked the written form of feedback. Our findings are comparable with the findings of a study conducted in Sidama zone, South Ethiopia (2). Planned supervision patterns will help to identify and fill the health facility gaps. Thus, it should be strong enough to support all facilities. From the drug and diagnostic supplies of structural quality of TB cares, all of the studied hospitals that had first line anti-TB drugs were sufficient for one month. This is consistent with the national minimum recommendation that very facility should have at least a one month stock level for existing patients.

Health education program for TB control activities was found to be poor in all health facilities, and there were no TB posters posted in visible areas. This is also comparable with a study conducted in Sidama zone and Addis Ababa, Ethiopia (2,11).This might be due to poor attention given by health care providers and program coordinators for the health education activities, even if it has a great role on prevention of TB transmission. Only one of the studied hospitals had clean water supply. This finding was in line with a study conducted in Sidama zone (2) but opposed to a study conducted in Jimma(8). This might be due to lack of resources or lack of attention for clean water supply near to TB clinic. The healthcare provider also prescribed appropriate regimen and was capable of assessing adherence. This is also in line with the study conducted in Jimma and Addis Ababa (2,11). This might be due to adequate access of training regarding the management of TB. This study also revealed that health care providers did not give respect to and approach the patients in a friendly manner. This finding is in line with a study conducted in Sidama (2). However, it is contrary to a study conducted in Addis Ababa (11). This might be due to difference in compassionate care between health professionals.

Patient satisfaction is an important quality outcome indicator of healthcare in the hospital settings. This study revealed that the overall level of patients satisfaction score with TB treatment services was 53.7%. The level of patients' satisfaction score in this study was comparable with a study conducted in public health facilities of Bahir Dar city administration where about 53.8% of respondents were fully satisfied with their TB treatment service (13). However, the level of patients' satisfaction found in our study was lower than the one in a study done in Sidama zone, which was 71.6% (2). This discrepancy might be due to variations in structural and process dimensions between the studied hospitals and other hospitals. In line with a study conducted in Addis Ababa (12), binary logistic regression analysis results of our study showed that having TB symptoms hada significant association with level of patients' satisfaction with TB care.

The findings of this study indicated that there was poor overall structural dimension of quality of TB care services being rendered to patients. However, each parameter used to measure structural dimension may not have equal contribution to the quality of care. This study also found that the overall level of patient satisfaction was low towards TB care. Having TB symptoms during TB treatment was the only statistically significant and independent predictor of the level of patient satisfaction. Thus, regular and continuous health education is needed to prevent unnecessary outcomes. In addition, continuous supportive supervision is important to identify the gaps, to conduct timely intervention and give necessary training for all healthcare providers who are working in TB clinics.
